# Research and Remedies

**Published:** 1912-08-24

**Authors:** 


### RESEARCH AND REMEDIES.

### How Intestinal Obstruction Causes Death.

Several hypotheses have been propounded to explain the rapid onset of fatal symptoms in cases of intestinal obstruction, but most of them have been founded upon doubtful inferences from complicated clinical phenomena rather than upon exact knowledge. In the *Johns Hopkins Hospital Bulletin* Stone, Bernheim, and Whipple narrate the result of some experimental work upon dogs, which leads them to the following conclusions. High "loop" obstruction is rapidly fatal to dogs; low loops (ileum) of similar nature are much less rapidly fatal. Surgical drainage of the loop will, however, save the animal's life; and total excision of the loop does not necessarily impair the animal's health. The secretion obtained from these loops is highly toxic, and causes profound splanchnic paralysis. When introduced into normal animals it produces many changes similar to those found in animals with closed duodenal loops—low blood pressure and temperature, excretion of large quantities of fluid into the intestinal canal, and fatal shock. The toxic material is not injured by heating to 60° C., by centrifuging, or by filtering; nor is it impaired by prolonged autolysis, by pancreatic digestion, or by bacterial fermentation. No such substance can be obtained from the normal intestinal mucose. The practical bearing of this research would seem to be direct confirmation of the value of puncturing and emptying any loop of distended intestine when relieving an acute obstruction, a process the utility of which most surgeons are already agreed upon.

### Unrecognised Typhus Fever.

Two or three years ago Dr. Brill, of New York, described a disease of which he had notes of 221 cases, but one that could not be allocated (in his opinion) to any of the diseases described in current text-books. He considered the possibility of this disease being a mild form of typhus fever, but rejected the idea on grounds which appeared to him to be sufficiently good. Since then many other clinicians have paid attention to "Brill's disease," and the view has been steadily prevailing that it is, after all, typhus fever, and not a separate clinical entity. The symptoms of these cases may be summarised briefly thus: incubation period of four to five days; sudden onset with severe headache and high pyrexia; a maculo papular eruption; a crisis at the end of nine to sixteen days of fever; and rapid recovery. It is obvious how closely this clinical picture resembles that of typhus fever, except for the much less dangerous and severe course of the disease. The view that the two diseases are really one is strongly supported by the experimental work of Anderson and Goldberger, who succeeded in infecting monkeys with both of them. They showed that a monkey which has had "Brill's disease" is immune to typhus, and that one which has had typhus is immune to "Brill's disease." It has long been known that typhus is a dirt disease, and there seem to be good grounds for believing that its contagion is conveyed by lice. Why it should be so benign in New York and Chicago is difficult to say.

